# Supplementary material for: Ribosome Structural Changes Dynamically Affect Ribosome Function
Source: Int J Mol Sci. 2024 Oct 17;25(20):11186. doi: 10.3390/ijms252011186 (PMC11508205; doi:10.3390/ijms252011186)
Supplement: Supplementary file 1 [file ijms-25-11186-s001.zip › ijms-3212674-supplementary.pdf]

Table S1. Comparison of amino acid sequence in paralogous r-proteins in *S cereviciae*

Paralogue pair	Protein encoded by paralogue pair	Diff in coding DNA sequence	Difference in aa sequence											
RPS0A/B	uS2	10%	F81Y	S128I	Q213V	V214A	A215E	F217A	T219A	T220A	A223G	G224E	A228V	E235G
RPS1A/B	eS1	8%	K11R	Q16L	R19K	S56N	I84V	K174R	G178N	T245S				
RPS4A/B	eS4	2%	**Nill**											
RPS6A/B	eS6	1%	**Nill**											
RPS7A/B	eS7	13%	A3V	P4V	T13S	Q22K	A23T	V25I	E26D	N29S	E39D	F42I	A51T	A59V
				I60L	S66A	A68S	G69A	F70Y	Q86P	I125V	L129M	E188Q	H190N	
RPS8A/B	eS8	2%	**Nill**											
RPS9AB	uS4	3%	K40R	D189E	D192E	E193-	A194-							
RPS10A/B	eS10	7%	E6Q	D7E	T98S									
RPS11A/B	uS17	4%	**Nill**											
RPS14A/B	uS11	8%	S2A	-D4	V5L									
RPS16A/B	uS9	6%	**Nill**											
RPS17A/B	eS17	5%	V137N											
RPS18A/B	uS13	5%	**Nill**											
RPS19A/B	eS19	5%	P2A											
RPS21A/B	eS21	9%	V51I											
RPS22A/B	uS8	10%	I26L											
RPS23A/B	uS12	3%	**Nill**											
RPS24A/B	eS24	3%	**Nill**											
RPS25A/B	eS25	8%	T105A											
RPS26A/B	eS26	8%	E106D	D113A										
RPS27A/B	eS27	15%	V62I											
RPS28A/B	eS28	12%	N3S											
RPS29A/B	uS14	8%	Y14F	I31V	G35D	N43H	N53H	F55Y						
RPS30A/B	eS30	5%	**Nill**											
RPL1A/B	uL1	1%	**Nill**											
RPL2A/B	uL2	3%	**Nill**											
RPL4A/B	uL4	1%	T356A											
RPL6A/B	eL6	9%	S6T	K5Q	L18P	A25V	I65V	S66T	N127T	A132T	I156L	M177L		
RPL7A/B	uL30	3%	A2S	A3T	S16T	V26I	S242A							
RPL8A/B	eL8	8%	T22A	R23K	V59L	V116I	A141S	V180I	N252T					
RPL9A/B	uL6	6%	V11I	T188V										
RPL11A/B	uL5	3%	A3T											
RPL12A/B	uL11	8%	**Nill**											
RPL13A/B	eL13	7%	T41A	N129D										
RPL14A/B	eL14	4%	A89G											
RPL15A/B	eL15	7%	Q11E	D153N										
RPL16A/B	uL13	13%	V3-	E4Q	G11A	G13D	V16L	V22T	V223I	L28V	E40A	F80L	L84I	V104I
				A158D	S163R	A179S	N182S	T184A	A186S	A196S	L196F			
RPL17A/B	uL22	8%	E49D	A183S										
RPL18A/B	eL18	5%	**Nill**											
RPL19A/B	eL19	6%	**Nill**											
RPL20A/B	eL20	5%	**Nill**											
RPL21A/B	eL21	6%	L27M	V33I										
RPL22A/B	eL22	21%	I11V	A12I	F15L	A34S	E43D	V54I	T55E	T61S	V62I	T68S	T98I	K99R
				T100Q	E102Q	R104K	A106V	-115A	E121D					
RPL23A/B	uL14	6%	**Nill**											
RPL24A/B	eL24	7%	I5V	K119R	T133V	S135G	F138V							
RPL26A/B	uL24	7%	Q6E											
RPL27A/B	eL27	10%	L51S											
RPL31A/B	eL31	8%	D58E											
RPL33A/B	eL33	9%	D40E											
RPL34A/B	eL34	7%	A119S											
RPL35A/B	uL29	1%	**Nill**											
RPL36A/B	eL36	11%	T2A	S19Q										
RPL37A/B	eL37	10%	A41S	Y47H	G50A	S84K	K85A	A86T						
RPL40A/B	eL40	6%	**Nill**											
RPL41A/B	eL41	1%	**Nill**											
RPL42A/B	eL42	2%	**Nill**											
RPL43A/B	eL43	10%	**Nill**											
RPP1A/B	P1A	30%	T3-	E4D	A6I	L7I	Y9F	L12F	S17A	E18G	I19L	S22T	E24D	K25N
				L29I	N31K	N34G	V35A	P36N	E38D	I40V	I44V	F45Y	D50E	Q52K
				N53D	D56E	L57I	V59S	N60G	-62H	S63N	A66P	A67V	P69G	V72A
				G74S	V76A	G78A	G79A	E80-	A81G	E83D	E85A	K88E	-90K	K95A
RPP2A/B	P2A	28%	N11V	A12Q	A13G	T16A	-17A	D19S	T21A	K22D	I26V	L27V	I32A	I34V
				E35D	D36E	E37A	K38R	V39I	S40N	S41E	V42L	A45S	-50G	V52L
				D53E	L55I	A57A	N60Q	E61K	L63F	A65T	A68T	A69G	P71A	A72S
				-75A	-76A	G78A	A80G	S83A	D85G	A86D	A97K			

Source https://www.yeastgenome.org/
